# Paternal Genetic Structure of Hainan Aborigines Isolated at the Entrance to East Asia

**DOI:** 10.1371/journal.pone.0002168

**Published:** 2008-05-14

**Authors:** Dongna Li, Hui Li, Caiying Ou, Yan Lu, Yuantian Sun, Bo Yang, Zhendong Qin, Zhenjian Zhou, Shilin Li, Li Jin

**Affiliations:** 1 Department of Biology, Hainan Medical College, Haikou, Hainan, China; 2 MOE Key Laboratory of Contemporary Anthropology, School of Life Sciences, Fudan University, Shanghai, China; 3 Department of Genetics, School of Medicine, Yale University, New Haven, Connecticut, United States of America; 4 Department of Anatomy, Third Military Medical University, Chongqing, China; University of Glasgow, United Kingdom

## Abstract

**Background:**

At the southern entrance to East Asia, early population migration has affected most of the Y-chromosome variations of East Asians.

**Methodology/Principal Findings:**

To assess the isolated genetic structure of Hainan Island and the original genetic structure at the southern entrance, we studied the Y chromosome diversity of 405 Hainan Island aborigines from all the six populations, who have little influence of the recent mainland population relocations and admixtures. Here we report that haplogroups O1a* and O2a* are dominant among Hainan aborigines. In addition, the frequency of the mainland dominant haplogroup O3 is quite low among these aborigines, indicating that they have lived rather isolated. Clustering analyses suggests that the Hainan aborigines have been segregated since about 20 thousand years ago, after two dominant haplogroups entered East Asia (31 to 36 thousand years ago).

**Conclusions/Significance:**

Our results suggest that Hainan aborigines have been isolated at the entrance to East Asia for about 20 thousand years, whose distinctive genetic characteristics could be used as important controls in many population genetic studies.

## Introduction

It is well-known that East Asians exhibit many uniquely derived characteristics in their genetic structure because the populations have been isolated from those of Western Eurasia for some time [1]. The origin of East Asians and their specific genetics features have been an area of great interest in the study of human population expansion into eastern Asia, which has been deeply discussed within the scientific community. However, the detailed timing and route of expansion of modern humans into eastern Asia remains controversial. As a south-north cline of East Asian's genetic structure was observed in various studies [2]–[4], both southern origin [5] and northern origin of East Asians [6] were supported by different researchers. Some even suggested that East Asians are a mixture of both southern and northern migrants. However, studies of the Y chromosome as a steady genetic material may help resolve the debate. Among the Y chromosome haplogroups studies, a few haplogroups showed a northern origin, linking East Asians to the Central Asian populations [7]. However, most of the haplogroups, especially the East Asian dominant haplogroup O [8], [9], appear to originate in the South of Eastern Eurasia [2], [10], [11]. Because southern migrants are dominant in the East Asian populations, it is important to realize that the southern entrance is important in the formation of the East Asian population. The southern entrance might be the border between China and Indo-China Peninsular countries (Myanmar, Laos, and Vietnam).

Unfortunately, many events of population relocation in the history of East Asian have deeply buried the original diversity pattern of populations around the south entrance to East Asia. The most notable of these events are the backward migrations of Han Chinese [4] and of Tibeto-Burman [8], [12] to South China and Southeast Asia. Most of the southern populations, even the populations in the Islands of Southeast Asia and New Guinea have been “disturbed” by these northern returnees [13]–[17]. Therefore, it is very hard to know the original genetic structure at the time when the populations entered East Asia and the different routes though which different Y haplogroups went. Isolated populations at the entrance to East Asia will be most helpful to resolve this question.

**Figure 1:**
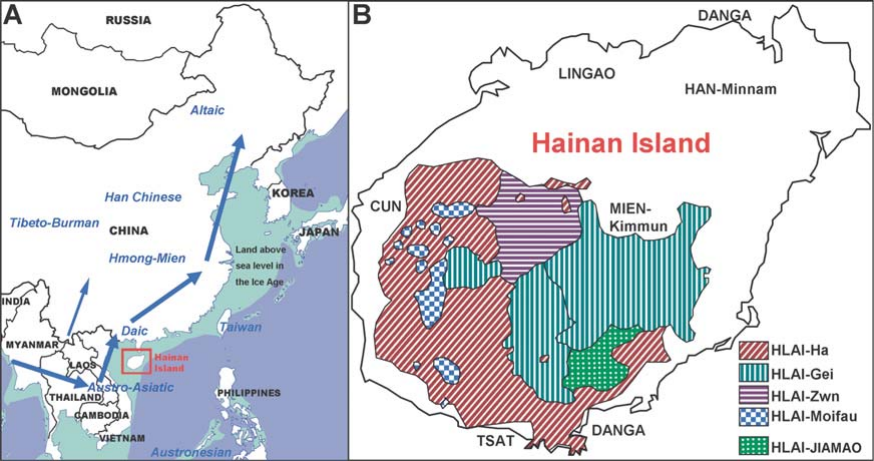
Geographic location of the Hainan Island and distributions of the Hainan aborigines. (A) The Hainan Island was at one of the entrances to East Asia during the earliest migration times of modern human. During this time, which coincides with the last Ice Age, the island was still connected with the mainland. The arrows on the map represent the possible routes for southern migrants into East Asia, though not precise. (B) Distributions of the Hlai branches and the populations around them. Cun is another kind of Hainan aboriginal population. Lingao is an archaic migrant population of Kam-Sui phylum. Tsat is an Austronesian population from South Vietnam. Mien and Han people came from the mainland recently. Danga is a fishing tribe living on the sea without any migration records.

Here we report that we found several isolated aboriginal populations on the Hainan Island in the southern tip of East Asia (Fig. 1A) that represent relatively ancestral and “undisturbed” Y chromosome genetic structures. We demonstrated that the paternal genetic structure of Hainan aborigines is apparently different from the mainland East Asian populations.

Hainan Island is a big island in the Tonkin Bay between East Asia and Southeast Asia. During the last Ice Age when the sea level was much lower than it is today, it was connected to the continent [18], [19], and lay on one of the ways of modern human migration from Southeast Asia to East Asia (Fig. 1A). The six Hainan aboriginal populations, living in the central and southern mountain areas of Hainan Island (Fig. 1B), may be the direct descendants of the original migrants. These aborigines are believed to have remained isolated for thousands of years since their island was separated from the continent by marine transgression eleven to seven thousand years ago [19]. The ethnic classification of Hainan aborigines exactly matches the linguistic classification [20]. The Hainan aborigines are classified into two groups, Hlai and Cun, both belonging to the most primordial branches of the Daic (also called Tai-Kadai) linguistic phylum, and also showing many resemblances to Malayans (under Austronesian phylum) in some respects[21]. Hlai has a population of more than 1.2 million (2000 census), and can be classified into five subgroups (Fig. 1B), showing high cultural diversity. All of the five subgroups, Ha, Gei, Zwn, Moifau, and Jiamao, have moderate population sizes. Even the smallest subgroup, Moifau, has a population of around 60,000. The languages of the Hlai subgroups are quite different and cannot be understood by each other [22]. Cun has a population of around 80,000, which exhibits very different linguistic and cultural characteristics from the Hlai. The history of Hainan aborigines is unknown as very few archaeological studies have been performed in Hainan. A finding in the *Luobidong* Cave of Sanya County revealed that modern humans may have lived in Hainan Island already about 10,000 years ago in Paleolithic Age [23], long before the relocation of other East Asian populations. The earliest Neolithic site of Hainan was the 6,000 years old *Dongfang Xinjie* Shell-heap site found in the present area of Cun people (confirmed by personal communication with Prof. Side Hao)[24], indicating the Neolithic package had arrived in Hainan before 6,000 years ago. Therefore, the Hainan aborigines may have maintained the genetic structure closest to the original ancestors of East Asians. However, there have not been any detailed genetic studies on Hainan aborigines, in contrast to the well-studied Taiwan aborigines [9], [17], [25] who are also isolated albeit rather far from the entrances to East Asia (either the southern entrance from Indo-China peninsula or the northern entrance from Central Asia).

## Results and Discussion

In our study, we analyzed the Y chromosome diversity of all the six populations of Hainan aborigines. We examined 22 single nucleotide polymorphisms (SNPs) and seven short tandem repeat (STR) polymorphisms in 405 male Hainan aborigines, and determined the haplogroups based on the nomenclature of YCC [26] and ISOGG [27]. We found that the population samples are very similar in the Y-SNP haplogroup frequencies (Table 1). The haplogroups O1 and O2 are most frequent in each population, and are most probably the original haplogroups of the Hainan aborigines. In one of the aboriginal populations, the Gei, the total frequency of these haplogroups reaches 100%, indicating that it is an extensively bottlenecked population. Actually, Gei locates in the most remote mountain area and may have developed from a much smaller population, which is supported by the low Y-STR diversity of the Gei sample (Table S1). Haplogroups O1 and O2 are also frequent in the indigenous populations of Taiwan Island and southmost areas of mainland East Asia; however, these two haplogroups are not as dominant as they are in the Hainan aborigines. The Austronesian populations on the south and east side of the South China Sea, Borneo and Philippines also have high frequency of O1 and O2 [9], [13], [17], consisting with the linguistic resemblance between Daic and Austronesian [21]. In the populations other than Daic and Austronesian, the frequencies of O1 are quite low, especially in the Austro-Asiatic populations, the indigenous group of western Indo-China peninsula [28], [29]. Haplogroup O3, which is common in Sino-Tibetan speaking populations [10] such as the Han (50.51%) and Tibeto-Burman (54.70%), is rare in Hainan aborigines (6.91%) while it is present in Taiwanese aborigines at a frequency of 11.36% (0%–37.6%) [17], [25], [28], and in the mainland southern (Daic 19.60%) and central (Hmong-Mien 54.02%) indigenous populations at a higher frequency [29]. This suggests that there has been less male admixture between the Han and the Hainan groups compared to the Taiwanese and mainland southern indigenous groups. Moreover, unlike in mainland populations, haplogroups D, P, N, and Q were absent altogether in Hainan aborigines.

**Table 1:** Y chromosome haplogroup frequencies.

Linguistics	Population	Sample size	Haplogroups (%)										
			C3*	F*	K*	O1a*	O2*	O2a*	O3*	O3a1	O3a3	O3a5*	O3a5a*
Hlai	Ha	74	4.05			22.97	5.41	59.46	5.41			1.35	1.35
Hlai	Moifau	66				28.79		66.67	3.03			1.52	
Hlai	Gei	62				8.06		91.94					
Hlai	Zwn	75			1.33	32.00	5.33	58.67	1.33		1.33		
Hlai	Jiamao	50	2.00	2.00		40.00		46.00	6.00		2.00		2.00
Kadai	Cun	78	5.13			57.69	17.95	3.85	8.97	1.28		3.85	1.28

**Figure 2:**
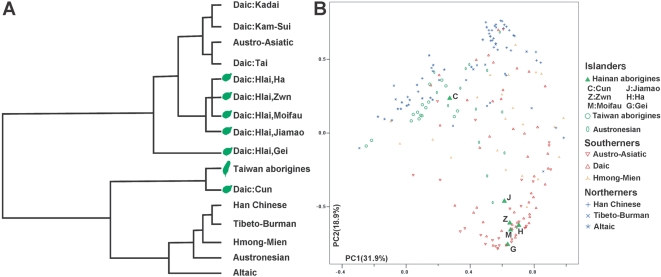
Clustering of Hainan aborigines and other East Asian ethnic groups. (A) Dendrogram of complete linkage exhibits that Hlai and Cun are different; each forms an outer clade of the cluster. (B) Principal component (PC) plot shows a clear south-north polarization. Hlai populations are all in the southern terminal with Dai and Austro-Asiatic populations. Cun is close to the Taiwan aborigines and the Austronesians on the northern side.

To determine the genetic relationship between Hainan aborigines and other populations, we performed two methods of clustering analyses (Fig. 2) using the Y-SNP haplogroup frequencies of Hainan aborigines and other East Asian populations [2], [4], [8]–[10], [12], [13], [28]. In the dendrogram of Fig. 2A, the East Asian populations are clustered into two groups: a southern group (Daic, etc.) and a northern group (Han, etc.). All the mainland Daic populations were clustered with the Austro-Asiatic population. Daic and Austro-Asiatic populations are scattered in mainland Southeast Asia (Fig. 1A), and their distributions overlap. We assumed that there must have been sufficient gene flow between them and classified them into one group as their Y-SNP frequencies are similar. The Hlai subgroups formed an outer clade of the southern group, and the bottlenecked and isolated subgroup of Hlai, the Gei, is on the most outside of the clade. These results indicate that the Hlai are quite different from the mainland mixed populations. In the northern group, Hmong-Mien, Han Chinese, and Tibeto-Burman were most similar to each other, while the Austronesian and Altaic were all influenced by the Sino-Tibetan populations genetically. We found the outer clade of this group to be Taiwan aborigines and Hainan Cun, exhibiting relatively low haplogroup diversity. It is, however, not clear why Cun was found to be similar to the Taiwan aborigines.

PC analysis also shows that the studied populations can be divided into a southern group and a northern group (Fig. 2B). Based on this PC plot, the Taiwan aborigines were closer to the northern group, and two kinds of Hainan aborigines are clustered into different groups. Cun can be placed into the northern group, together with the Taiwan aborigines. All of the Hlai populations were very close to the southern terminal of the south-north division, re-exhibiting their isolated genetic structures. This division is especially true for the Gei in the south end of the division.

**Figure 3:**
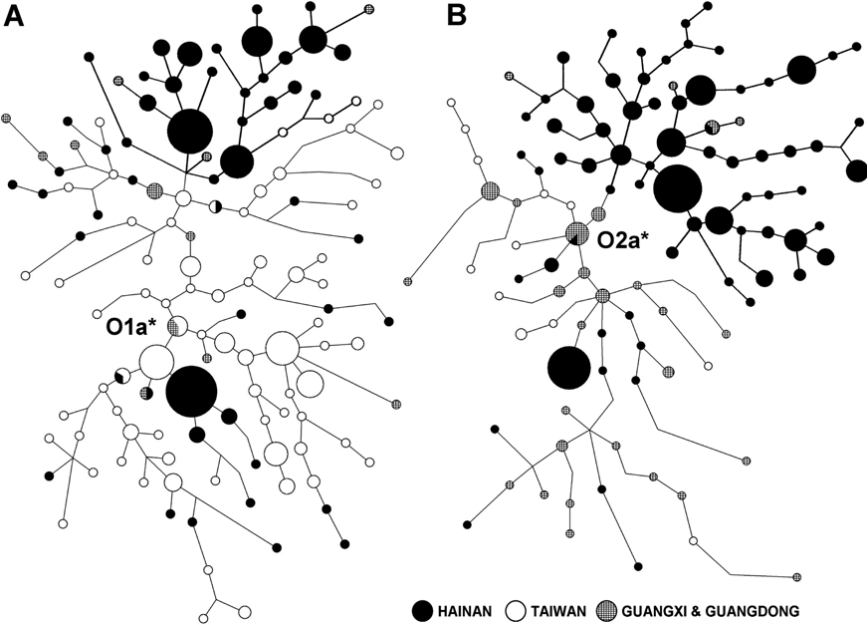
Y chromosome STR networks. As the original networks were too complicated to display, here we presented the shortest trees of the largest possibility reduced from the networks. The original networks are in Dataset S1. In each of the trees, Hainan aborigines occupy a fairly exclusive clade (in bold lines), indicating their long history of isolation.

According to the SNP analyses, Hainan aborigines have been isolated from the northern populations, especially the Han Chinese migrations into South China who have brought the high frequency of haplogroup O3. The southward migration of Hmong-Mien populations might also have contributed to the increase in the frequency of O3 in South China. Our recent study revealed that Hmong-Mien and Sino-Tibetan may have a most recent common ancestor in southwestern China, and the O3 haplogroup was dominant in their common ancestral population (Unpublished data of Li Jin). As we have mentioned, O3 is almost absent in Hainan aborigines. However, the dominant haplogroups of Hainan aborigines, O1 and O2, are also dominant in mainland Daic populations and Taiwan aborigines. The SNP haplogroup analyses cannot exclude the gene flow between Hainan aborigines and Daic-Taiwan populations. Thus, aside from the dendrogram and the PC plot of SNPs, we analyzed the STR networks (Fig. 3 and Dataset S1) of the two major haplogroups, O1a* and O2a*, among the samples from the Hainan aborigines, Taiwan aborigines, and mainland Daic.

In these networks, reference data [28] were from Taiwan aborigines and the Daic population of Guangxi and Guangdong, two provinces north to Hainan. In the top part of each network of Figure 3, Hainan aborigines (shown in black nodes) formed an almost exclusive clade (shown in bold lines) with few individuals from other populations, suggesting that Hainan aborigines had been isolated from other Daic populations and Taiwan aborigines. Furthermore, most of the Hlai haplotypes were in the top clades, while Cun haplotypes formed two smaller clades in both networks (the black nodes in the lower part of the networks). The size of the Hlai clade is relatively large, occupying nearly one third of the network. In the O2a* network, the Hlai clade is much larger than the mainland or Taiwan part, suggesting that Hlai is much older than the mainland Daic or Taiwan aborigines, not a derived group of mainland Daic. It took quite long time for the STRs to mutate and form this large size of clade. We estimated the population ages to be around 36 thousand years for O1a*, around 19 thousand years for the Hainan clade in the O1a* network, and around 32 thousand years for O2a*, and around 26 thousand years for the Hainan clade in the O2a* network (Table 2). Please note that our estimate for O2a* was based on the South China populations, and therefore differs from the age of O2a* of the world population. As these two haplogroups are nearly absent in North China, the ages in South China are most probably the time for these two haplogroup to enter East Asia. The ages of O1a* and O2a* are close, however, falling between 18 to 26 thousand years. This time frame corresponds to the peak time of the last Ice Age (around 20 thousand years ago) [18], when the continental shelves in the China Seas were above sea level and provided a short cut for modern humans to enter East Asia.

**Table 2:** Age estimates for haplogroups O1a* and O2a* (thousand years).

	NETWORK		BATWING	
	Age	S.E.	Age	95% C.I.
O1a*	36.1	7.0	39.5	22.9–75.3
O1a* Hainan	18.9	5.6	14.5	7.2–33.0
O2a*	31.7	7.3	30.4	20.8–53.5
O2a* Hainan	25.7	7.6	19.6	11.3–44.5

The effective population size used in BATWING is 1300.

There might have been more than one route for early migrants of modern human into East Asia. Another possible route of entering East Asia might be on the west side of the Southeast Asia-East Asia border (from Myanmar to Yunnan and inland China, Fig. 1A). We assumed that O3 haplogroups were carried by the Sino-Tibetan and Hmong-Mien ancestors through this western route, though there have not been enough evidence (Our recent investigations on Hmong-Mien and Mon-Khmer populations gave a clue to the possible western route). However, O3 could also have emerged in the same and possibly single eastern route but later after the time of emergence of O1a* and O2a*. The O3 haplogroups subsequently influenced most of the other populations through relocations. According to absence of O3 in the isolated populations of Hainan Island, O3 was not carried through the entrance around Hainan when ancestors of Daic people first arrived in East Asia, however, establishing O2a* to be one of the oldest haplogroups (more than 40 thousand years, unpublished data of Li Jin) carried by the earliest migrants into East Asia along this eastern route. The haplogroup O2a* might also be the first one that arrived in Hainan. The age of the Hainan clade of O2a* was determined to be around 26 thousand years, and is much older than the dating results of the *Luobidong* Cave site, the oldest archaeological finding in Hainan. We propose this age to be that of the Hainan aborigines, who have been essentially isolated since. Earlier archaeological sites may be found in Hainan Island in the future.

Our analysis of the STR network of O1a* shows that the Taiwan nodes are closer to the center of the network than the Hainan nodes, indicating that Taiwan was closer to the origin of this haplogroup geographically. Therefore, O1a* might have originated to the east of Hainan Island, and flowed back to Hainan subsequently, establishing the age of the Hainan clade in O1a* to be a little younger than that in O2a*. O1a* was also found in the 5,000-year-old Neolithic human samples from the east coastal area of China [29]. The Neolithic period of East Asia began around 8,000 years ago. It is possible that O1a* might have diffused along with the Neolithic cultures to the Hainan Island. The age of O1a* in Hainan (around 18 thousand years) is older than that of the earliest Neolithic site found in Hainan (around 6,000 years)[24]. A possible explanation could be that the age was counted from the O1a* people departed from the ancestral O1a* group (maybe the ancestor of Taiwan aborigines) before they arrived in Hainan. We calculated the divergence time between O1a* of Taiwan and Hainan aborigines to be 22.0 (95% CI: 12.5–46.8) thousand years.

In conclusion, our findings indicate that Hainan aborigines descend from early migrants who entered East Asia during the last Ice Age, and have been isolated since then. The Hainan aborigines have hardly been influenced by the population relocations in mainland East Asia, and the Y-SNP haplogroup patterns of the Hainan aborigines are closest to the original genetic structure of the early migrants. We suggest that Hainan aborigines cannot only be used to reveal the origin of East Asians and their unique genetic features, but that they can also serve as a model for East Asian population genetic studies.

As we know, isolated populations are most helpful in some genetic studies. For example, the nosogenetic factors of the complex diseases are apparently reduced in the isolated populations and will be much easier to analyze. Furthermore, with relatively big populations and long history, most of the Hainan aborigines have unlikely undergone genetic drift, and developed relatively high Y-STR diversity beside their low Y-SNP diversity. It is believed that Hainan aborigines could also have higher diversity of autosomal variances than the other East Asians, and lower linkage disequilibrium, which is more valuable for the disease association studies to avoid the false positive results caused by high linkage disequilibrium. Therefore, we suggest more genetic studies to be done in the Hainan aborigines as a model of isolated East Asian population.

## Materials and Methods

Population samples were collected by drawing blood from the upper arms of 405 male volunteers of Hainan aborigines. The sample size of each population was given in Table 1. The locations of the populations were illustrated in Figure 1B. More detailed population information can be found online (http://www.ethnologue.com) by searching the ISO639-3 code [lic], [jio], and [cuq]. The healthy donors were from different villages and had different surnames, ensuring that the individual samples were unrelated. Written informed consents were signed by all the 405 volunteers. Our study of human blood was approved by the Ethics Committee of the Chinese National Human Genome Center at Shanghai.

Fifteen single nucleotide polymorphisms (SNPs) in the Y chromosome non-recombining portion were typed in the collected samples by PCR-RFLP (M130, M89, M9, M45, M120, M119, M110, M101, P31, M95, M88, M122, M164, M159, and M7). Six SNPs (M210, M208, M48, M8, M217, and M356) were typed by Taqman (Applied Biosystems Co.). Seven SNPs (Yap, M15, M175, M111, M134, M117, M121) and seven short tandem repeat (STR) polymorphisms (DYS19, DYS389I, DYS389II, DYS390, DYS391, DYS392, DYS393) were typed by using fluorescently labeled primers for PCR amplification. Denatured products were separated by acrylamide gel electrophoresis through the use of an ABI 3100 genetic analyzer to distinguish the alleles. These SNP and STR markers are all highly informative for studies of East Asian populations [8]–[10], [25], [28]. Y chromosome haplogroups were determined according to the classification of the Y-DNA Haplogroup Tree 2007 provided by International Society of Genetic Genealogy [27] developed from the nomenclature of Y Chromosome Consortium [26].

Dendrogram clustering and principal component (PC) analyses were performed by software SPSS13.0. STR median-joining networks were drawn by Network 4.201 [30], and ages were estimated in the networks according to the mutation rates estimated by Zhivotovsky et al. (6.9×10^−4^ per 25 years) [31]. In the age estimation, the total mutation rate was 1.932×10^−4^ per year, the sum of the seven STRs. We assumed an average of 25 years per generation, resulting in 5176 years per mutation in the networks. Time estimates were confirmed by BATWING [32].

## Supporting Information

Table S1 The Y-STR haplotype frequencies of the Hainan aborigines.
(0.04 MB XLS)

Dataset S1 The original networks of O1a* and O2a*.
(0.01 MB ZIP)
